# Efficient reference-free adaptive artifact cancellers for impedance cardiography based remote health care monitoring systems

**DOI:** 10.1186/s40064-016-2461-5

**Published:** 2016-06-17

**Authors:** Madhavi Mallam, K. Chandra Bhushana Rao

**Affiliations:** Department of Electronics and Communication Engineering, Jawaharlal Nehru Technological University, Kakinada, AP 533003 India; Department of Electronics and Communication Engineering, JNTUK, University College of Engineering, Vizianagaram, AP 535002 India

**Keywords:** Artifact canceller, Cardiovascular diseases, Impedance cardiogram, Non-negative algorithm, Remote health care

## Abstract

In this paper, a new model for adaptive artifact cancelation in impedance cardiography (ICG) signals is presented. It is a hybrid model based on wavelet decomposition and an adaptive filter. A novel feature of this model is the implementation of reference-free adaptive artifact cancellers (AAC). For this implementation, the reference signal is constructed using a wavelet transformation. During critical conditions the filter weights may be negative and cause an imbalance in the convergence. To overcome this problem, we introduce non-negative adaptive algorithms in the proposed artifact canceller. To accelerate the performance of the AAC, we propose exponential non-negative and normalized non-negative algorithms to update the filter coefficients. The computational complexity of the filtering section in a remote health care system is important to avoid inter-symbol interference of the incoming samples. This can be achieved by combining sign-based algorithms with the adaptive filtering section. Finally, several AACs are developed using variants of the non-negative algorithms and performance measures are computed and compared. All of the proposed AACs are tested on actual ICG signals. Among the AACs evaluated, sign regressor normalized non-negative LMS (SRN^3^LMS) based adaptive artifact canceller achieves highest signal to noise ratio (SNR). The SNR achieved by this algorithm in baseline wander artifact elimination is 8.5312 dBs, in electrode muscle artifact elimination is 7.5908 dBs and in impedance measurement artifact elimination is 8.4231 dBs.

## Background

Cardiovascular disease (CVD) refers to a large number of medical conditions relating to heart functionality. The World Health Organization (WHO) states that approximately 50 % of all deaths from non-communicable diseases (NCDs) are from CVDs (World Health Organization 2015a). Among these, most of the deaths are outside the hospital because the patient is not treated in a timely manner. Additionally, the American Heart Association reported that more than 1 in 3 have more than one type of CVD (American Heart Association 2015) and that CVDs are the number 1 cause of death globally. In this scenario, WHO has planned to reduce the deaths from NCDs by 25 % globally by 2025 (World Health Organization 2015b). Hence, research on cardiovascular health care technology is becoming intensely active. Among various methods of cardiac activity study, impedance cardiography (ICG) is a promising technique. ICG facilitates non-invasive and continuous monitoring of haemodynamic entities such as stroke volume (SV) and cardiac output (CO) in clinical scenarios. ICG measures the change in impedance that exists at the thorax from the physical activity of the heart muscle (Woltjer et al. 1997; Brown et al. 1994; Nagel et al. 1989). The methodologies and mathematical analyses used to calculate the ICG, SV and CO can be found in the literature (Scherhag et al. 2005; Kubicek et al. 1966; Sramek 1983; Kubicek et al. 1974; Bernstein 1986). Analysis of the ICG signal is an important task when treating a cardiac patient in critical conditions. However, during acquisition, the ICG signal encounters physiological and non-physiological artifacts. The artifacts include the baseline wander artifact (BWA), the electro-muscle artifact (EMA) and the impedance mismatch artifact (IMA). These artifacts cause changes in both the signal shape and tiny features, which are key parameters for clinical diagnoses. Therefore, to achieve high-resolution ICG signals for clinical investigations, the artifacts must be eliminated. Because most of the biomedical physiological and non-physiological phenomena are non-stationary, adaptive filtering techniques are likely to be a good remedy for this application. Adaptive filters can update their filter weights automatically to fit the input noise level. Several researchers have presented contributions on the analysis of ICG using signal processing techniques (Wang et al. 1995; Muzi et al. 1985; Ishiguro et al. 2006; Barros et al. 1995; Dromer et al. 2009; Yamamoto et al. 1988; Javaid et al. 2015; Sebastian et al. 2011). In these contributions, both least mean square (LMS) and recursive least square (RLS) algorithms are used. In a real-time clinical environment, and in critical conditions from an abnormal heart rhythm, the filter weights may be negative. The negative weights cause an imbalance in the convergence, resulting in poor filtering capability. To overcome this problem, we introduce non-negative adaptive algorithms in the proposed artifact canceller. To accelerate the performance of the AAC, we propose exponential non-negative and normalized non-negative algorithms to update the filter coefficients. The computational complexity of the filtering section in a remote health care system is important to avoid inter-symbol interference of the incoming samples. This can be achieved by combining sign-based algorithms with the adaptive filtering section. The remedy for unbalanced convergence and poor filtering performance of the algorithm is a modified LMS algorithm, in which a diagonal vector of the input is introduced in the weight update equation, i.e., a non-negative LMS (*N*^*2*^*LMS*) algorithm (Chen et al. 2011). This *N*^*2*^*LMS* keeps the filter weights from becoming negative from the abnormal rhythms of the heart. To improve the performance of the AAC, the *N*^*2*^*LMS* algorithm is varied, resulting in an exponential *N*^*2*^*LMS* (*eN*^*2*^*LMS*) and a normalized *N*^*2*^*LMS* (*N*^*3*^*LMS*) (Chen et al. 2014a, b). In conventional AACs, a reference signal, which is correlated with the noise component in the contaminated signal, is required (Thakor and Zhu 1991; Rahman et al. 2011; Karthik et al. 2013; Rahman et al. 2013). However, in a clinical environment, it is difficult to find a correlated reference. That is, the reference signal and the actual contaminated artifact in the ICG are not correlated. Therefore, a strategy using a discrete wavelet transformation (DWT) is implemented to construct a reference signal based on the contamination present in the actual signal (Peng et al. 2013). The AAC can then track the changes in the input signal, and, using wavelet decomposition methodology, automatically construct the reference signal. The reference signal is then utilized by the adaptive algorithm in the AAC to update the filter weight coefficients. In remote health care systems, computational complexity is also a factor that plays an important role when developing a lab on chip (LOC) or a system on chip (SOC) in modern health care telecardiology systems. Low complexity is desirable when constructing wearable and nano devices. In addition, if the computational complexity is large, the impulse response length of the receiver filters increases and thus the size of the filter increases. This cause inter-symbol interference at the input of the filter (Rahman et al. 2012). Therefore, to minimize computational complexity, and thus improving the suitability of the proposed AAC for remote health care systems, we combine the non-negative algorithms with the three simplified algorithms (Farhang-Boroujeny 1998). The simplified algorithms based on LMS recursion are known as sign regressor LMS (SRLMS), sign error LMS (SELMS) and sign sign LMS (SSLMS) algorithms. To reduce the computational complexity of the proposed algorithms, we combine the *eN*^*2*^*LMS* and the *N*^*3*^*LMS* algorithms with SRLMS, SELMS and SSLMS, resulting in the *SReN*^*2*^*LMS*, *SEeN*^*2*^*LMS*, *SSeN*^*2*^*LMS*, *SRN*^*3*^*LMS*, *SEN*^*3*^*LMS* and *SSN*^*3*^*LMS* algorithms. Based on these algorithms, we use the wavelet decomposition method to develop several AACs to eliminate artifacts from ICG signals. Wavelet decomposition technique is particularly useful in health care applications; where accurate knowledge of the noise may not be available. The performance of these AACs is compared using the signal-to-noise ratio. The theory, the analysis of the algorithms and the simulation results of the various implementations are presented in the sections that follow.

## Methods

**Fig. 1:**
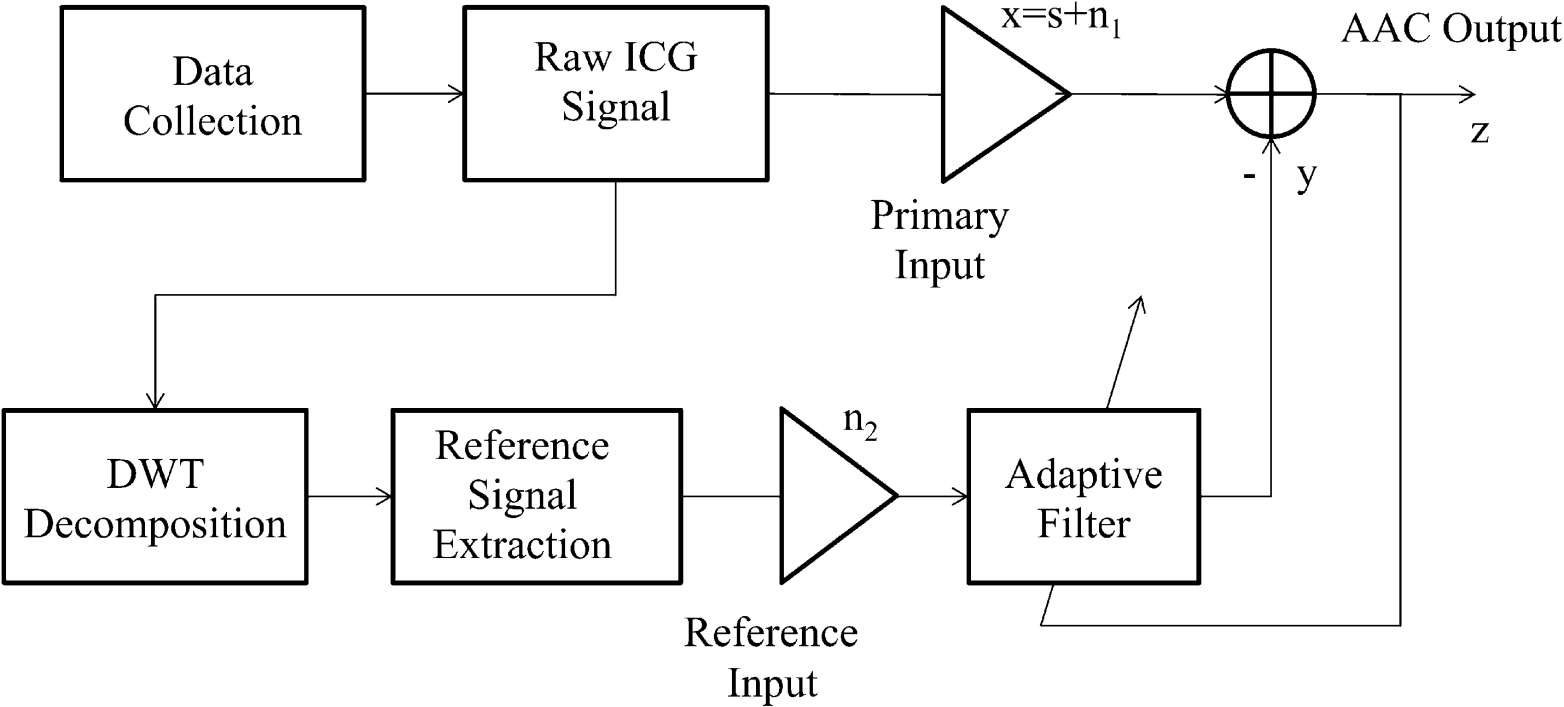
Block diagram of wavelet-based adaptive artifact canceller for ICG signal analysis

In this paper, we introduce a new technique of artifact cancelation in ICG signals for remote health care monitoring systems. During signal acquisition in a typical ICG remote health care monitoring system, some physiological and non-physiological contaminants add to the actual heart activity, leading to ambiguous diagnoses and measurements. In addition to these contaminants, channel noise also masks the tiny features of the ICG signal. The major artifacts encountered with heart activity are the baseline wander artifact (BWA), the electro-muscle artifact (EMA) and the impedance mismatch artifact (IMA). The BWA is a base-line drift of the ICG signal from respiration activity. The EMA is caused by muscle activity, and the IMA is caused by an impedance mismatch between the electrodes and the skin, or from a mismatch of the electrodes. At the receiving end, a clear high-resolution signal is required to present to the doctor for diagnosis. In this context, AAC plays an important role. Figure 1 shows a block diagram of a wavelet-based AAC for remote health care monitoring systems.

The recorded ICG signal with artifact contaminants is expressed as follows:

$$ICG(n) = s(n) + n_{1} (n)$$

where *ICG*(*n*) is the recorded ICG signal; *s*(*n*) is the original ICG signal generated from heart activity; and *n*_1_(*n*) is the artifact component (BWA or EMA or IMA or any combination of these three). In a remote system, *n*_1_(*n*) also includes channel noise.

The basic working principle of the proposed AAC is the following. The raw signal *ICG*(*n*) is input to the DWT decomposition unit. Using decomposition, a reference signal is constructed for any type of contamination present in the raw input ICG signal. The constructed reference signal is used as the reference signal for the adaptive algorithm to update its weight coefficients. The proposed AAC thus plays a vital role in the implementation of an intelligent remote health care monitoring system that is reference-free by constructing the reference signal itself from the contaminated input signal.

### Construction of the reference signal from the noisy input signal

**Fig. 2:**
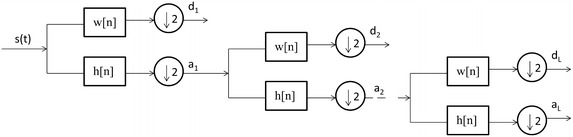
*L*-level decomposition of an ICG signal

The wavelet transform is used for signal decomposition in our model. It provides the temporal information for the signals whose frequency components are changing with time. The wavelet decomposition is a process of separating the signal into spectrally non-overlapping components. There are two categories of wavelet decomposition: continuous wavelet transforms (CWT) and discrete wavelet transforms (DWT). The CWT for a signal *s*(*n*) is as follows:

1 $$CWT\left({a,b} \right) = \mathop \int \nolimits_{- \infty}^{\infty} s\left(t \right)\frac{1}{\sqrt a}\varphi \left({\frac{t - b}{a}} \right)dt$$

where *a* and *b* are the scaling and shifting parameters, respectively, and $\varphi$(.) is the mother wavelet function. However, evaluating the scaling (*a*) and shifting (*b*) parameters for all possible scales is a computationally in feasible task. One possible way of solving the problem is choosing *a* and *b* as powers of two, in which case the DWT is as follows:

2 $$DWT\left({a,b} \right) = \frac{1}{{\sqrt {2^{l}}}}\mathop \int \nolimits_{- \infty}^{\infty} s\left(t \right)\varphi \left({\frac{{t - 2^{l} m}}{{2^{l}}}} \right)dt$$

where the scaling and shifting parameters are replaced by 2^*l*^ and *m*2^*l*^, respectively. Figure 2 shows the *L*-level wavelet decomposition of a signal *s*(*n*). In this scheme, the signal *ICG*(*n*)first passes through the LP and HP filters, whose cut-off frequencies are one-fourth of the sampling frequency *f*_*s*_ and down-sampled by 2, thus yielding an approximation *a*_*1*_ and detail *d*_*1*,_ which are coefficients of the first level. The same procedure is employed on the first level of the approximation coefficients*a*_*1*_, yielding the second level of approximation and detail coefficients. In this decomposition process, because of the down-sampling, the time resolution is halved and the frequency repulsion is doubled from the filtering operation. The frequency content of the signal at the *i*th level decomposition is given by $0 - f_{s} /2^{i + 1}\,\, {\text{and}}\, 0 - f_{s} /2^{i} , i = \left\{ {1,2, \ldots ,L} \right\}$ (Coifman and Donoho 1995; Percival and Walden 2000).

### Non-negative LMS-based algorithms for AACs

In the proposed AAC, the input is the raw contaminated ICG signal and the reference is the signal constructed from the DWT decomposition of the raw ICG signal. This process is shown in Fig. 1. The AAC consists of an FIR filter of length L taps. The weight coefficients are updated based on the weight update mechanism of various algorithms. The weight update mechanism for the basic LMS algorithm is as follows,

3 $$\varvec{W}\left({n + 1} \right) = \varvec{W}(n) + \eta \varvec{r}(n)e(n),$$

where *W*(*n* + 1) is the next weight coefficient; *W*(*n*) is the previous weight coefficient; *η* is the step size; *r*(*n*) is the reference signal, which is constructed from the DWT decomposition, required for training to eliminate noise from the raw signal *ICG*(*n*); and *e*(*n*) is the error generated, which is used as a feedback to the adaptive algorithm.

Because of the abnormalities in the ICG signal, i.e., the drastic variations in the signal features, the weight coefficients may become negative. This leads to poor performance of the adaptive algorithm in terms of convergence, stability and filtering capability. To overcome this drawback, a non-negative LMS (*N*^2^*LMS*) algorithm is proposed (Chen et al. 2011). The weight update mechanism is as follows:

4 $$\varvec{W}\left({n + 1} \right) = \varvec{W}(n) + \eta \varvec{D}(n)\varvec{r}(n)e(n),$$

where *D*(*n*) is the diagonal matrix of the weight coefficients *W*(*n*). The elaborated theory and analysis of *N*^*2*^*LMS* is presented by Chen et al. (2011).

In Eq. (4), each component of *W*(*n* + 1) is viewed as a variable step because of the combination of *ηD*(*n*). In the *N*^*2*^*LMS* algorithm, when the weights tend to zero, the convergence becomes unbalanced and the algorithm may diverge, causing the AAC to be ineffective for noise removal. To avoid the convergence imbalance characteristics in abnormal conditions, the exponential form *N*^*2*^*LMS* (*e N*^*2*^*LMS*) is proposed. The weight update mechanism is then as follows:

5 $${\mathbf{W}}\left({n + 1} \right) = {\mathbf{W}}(n) + \eta {\mathbf{r}}(n)e(n)W^{\gamma} (n)$$

For 0 < *γ* < 1, the *n*th weight update in Eq. (5) is larger than that in Eq. (4), which accelerates convergence towards the steady state error. Another direct way to accelerate the convergence of *N*^*2*^*LMS* is normalization with respect to data. The normalized *N*^*2*^*LMS* (*N*^*3*^*LMS*) is mathematically expressed as follows:

6 $${\mathbf{W}}\left({n + 1} \right) = {\mathbf{W}}(n) + \eta (n){\mathbf{D}}(n){\mathbf{r}}(n)e(n)$$

where *η*(*n*) is a variable step size with respect to the reference input as follows:

7 $$\eta (n) = \frac{\eta}{{\alpha + r^{t} (n)r(n)}}$$

where *α* is a small constant used to avoid numerical difficulties. The elaborated theory and analysis of the *eN*^*2*^*LMS* and *N*^*3*^*LMS* algorithms are presented in the literature (Chen et al. 2014a, b).

To minimize the computational complexity of the above algorithms, and hence to make them suitable for remote health care applications, we combine the *eN*^*2*^*LMS* and *N*^*3*^*LMS* algorithms with the simplified algorithms described by Farhang-Boroujeny (Farhang-Boroujeny 1998). The weight update mechanism equations for the *eN*^*2*^*LMS* algorithm variants then become the following:

1. The sign regressor version of the *eN*^*2*^*LMS* algorithm uses the following weight update equation:
  8 $$\varvec{W}\left({n + 1} \right) = \varvec{W}(n) + \eta \;sign\;\left({\varvec{r}(n)} \right)e(n)W^{\gamma} (n)$$

This algorithm is the sign regressor *eN*^*2*^*LMS* (*SReN*^*2*^*LMS*) algorithm. The major advantage of this algorithm is its low computational complexity in terms of multiplications, independent of filter length. To compute Eq. (8), only one multiplication is required. Another important feature of the sign regressor (SR) algorithm is that its convergence characteristics are only slightly inferior to those of its normal version. This is caused by the normalization involved in the signum function (Farhang-Boroujeny 1998; Eweda 1990; Koike 1999).

2. The sign error version of the *eN*^*2*^*LMS* algorithm uses the following weight update equation:
  9 $$\varvec{W}\left({n + 1} \right) = \varvec{W}(n) + \eta \varvec{r}(n)sign\left({e(n)} \right)W^{\gamma} (n)$$

This algorithm is the sign error *eN*^*2*^*LMS* (*SEeN*^*2*^*LMS*) algorithm.

3. The sign sign version of the *eN*^*2*^*LMS* algorithm uses the following weight update equation:
  10 $$\varvec{W}\left({n + 1} \right) = \varvec{W}(n) + \eta sign\left({\varvec{r}(n)} \right)sign\left({e(n)} \right)W^{\gamma} (n)$$

This algorithm is the sign sign *eN*^*2*^*LMS* (*SS eN*^*2*^*LMS*) algorithm.

Similarly, the weight update mechanism equations for the *N*^*3*^*LMS* algorithm variants are written as follows:

1. The sign regressor version of the *N*^*3*^*LMS* algorithm uses the following weight update equation:
  11 $$\varvec{W}\left({n + 1} \right) = \varvec{W}(n) + \eta (n)\varvec{D}(n)sign\left({\varvec{r}(n)} \right)e(n)$$

This algorithm is the sign regressor *N*^*3*^*LMS* (*SRN*^*3*^*LMS*) algorithm.

2. The sign error version of the *N*^*3*^*LMS* algorithm uses the following weight update equation:
  12 $$\varvec{W}\left({n + 1} \right) = \varvec{W}(n) + \eta (n)\varvec{D}(n)\varvec{r}(n)sign\left({e(n)} \right)$$

This algorithm is the sign error *N*^*3*^*LMS* (*SEN*^*3*^*LMS*) algorithm.

3. The sign sign version of the *N*^*3*^*LMS* algorithm uses the following weight update equation:
  13 $$\varvec{W}\left({n + 1} \right) = \varvec{W}(n) + \eta (n)\varvec{D}(n)sign\left({\varvec{r}(n)} \right)sign\left({e(n)} \right)$$

This algorithm is the sign sign *N*^*3*^*LMS* (*SSN*^*3*^*LMS*) algorithm.

In Eqs. (11)–(13), during the normalization process, *r*^*t*^(*n*)*r*(*n*), in the denominator of *η*(*n*), requires *L* multiplications. To minimize the number of multiplications, we use only the maximum value of *r*(*n*) instead of using all *L* values. The new *η*(*n*)is *η*_*m*_(*n*), as follows:

14 $$\eta_{m} (n) = \frac{\eta}{{\alpha + r_{m}^{t} r_{m}}}$$

The new weight update mechanisms for *N*^*3*^*LMS* and its three signed variants are then as follows:

15 $${\mathbf{W}}\left({n + 1} \right) = {\mathbf{W}}(n) + \eta_{m} (n){\mathbf{D}}(n){\mathbf{r}}(n)e(n)$$

16 $${\mathbf{W}}\left({n + 1} \right) = {\mathbf{W}}(n) + \eta_{m} (n){\mathbf{D}}(n)sign\left({{\mathbf{r}}(n)} \right)e(n)$$

17 $${\mathbf{W}}\left({n + 1} \right) = {\mathbf{W}}(n) + \eta_{m} (n){\mathbf{D}}(n)sign\left({{\mathbf{r}}(n)} \right)sign\left({e(n)} \right)$$

18 $${\mathbf{W}}\left({n + 1} \right) = {\mathbf{W}}(n) + + \eta_{m} (n){\mathbf{D}}(n)sign\left({{\mathbf{r}}(n)} \right)sign\left({e(n)} \right)$$

**Fig. 3:**
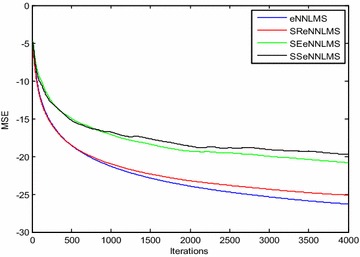
Typical convergence curves for ICG signal enhancement in a Gaussian environment using exponential non-negative LMS (eN^2^LMS) and its variants

**Fig. 4:**
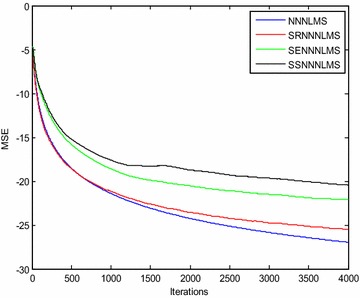
Typical convergence curves for ICG signal enhancement in a Gaussian environment using normalized non-negative LMS (N^3^LMS) and its variants

Figures 3 and 4 show the convergence curves of the *eN*^*2*^*LMS* and the *N*^*3*^*LMS* algorithms and their SR, SE and SS variants. The data in these figures show that *SReN*^*2*^*LMS* is only slightly inferior to the *eN*^*2*^*LMS*-based AAC, at the cost of a reduced number of multiplications. Hence, in practical implementations, if choosing between the *SReN*^*2*^*LMS* and the *eN*^*2*^*LMS* algorithms, the SR version is preferred. Similarly, between the *SRN*^*3*^*LMS* and the *N*^*3*^*LMS* algorithms, *SRN*^*3*^*LMS* is slightly inferior to *N*^*3*^*LMS,* but uses a reduced number of multiplications. Therefore, for real-time applications, the *SRN*^*3*^*LMS* algorithm-based AAC can be used. *N*^*3*^*LMS* is slightly faster converging than *eN*^*2*^*LMS,* as shown in Figs. 3 and 4.

## Results

In our experiments, we use the ICG signals obtained from a VU-AMS ambulatory system (Goedhart et al. 2006; Riese et al. 2003; Willemsen et al. 1996). ICG signals with different artifacts are included in our simulations. We use five ICG signals with 5000 samples: Data1, Data2, Data3, Data4 and Data5. In our simulation results, we show 2000 samples to illustrate the high-resolution signals. To evaluate the performance of the algorithms discussed above, we develop various AACs using the LMS, *eN*^*2*^*LMS*, *SR eN*^*2*^*LMS*, *SE eN*^*2*^*LMS*, *SS eN*^*2*^*LMS*, *N*^*3*^*LMS*, *SR N*^*3*^*LMS*, *SE N*^*3*^*LMS* and *SS N*^*3*^*LMS* algorithms. According to our proposed model, from the raw ICG signal, we construct a reference signal using DWT decomposition and use it as the reference in the adaptive algorithm. Using the above algorithms, we develop various AACs and calculate the signal-to-noise ratio (SNR), used as a measure of performance in our experiments. Comparisons of the SNR from the various algorithms are shown in Table 1. In addition to SNR measurements, we also tabulated the weight coefficients used to enhance Data1 during various artifact cancelations to examine the non-negative constraints of the non-negative algorithms, as shown in Table 2. The data in Table 2 show that the non-negative algorithms keep the weights from becoming negative. In our simulations, the filter length is 10 and the step size is 0.1. Because of space constraints, only the simulation results from Data1 are shown in this paper. ICG signals contaminated with BWA, EMA and IMA are used to illustrate the enhancement process.

**Table 1:** Comparison of signal to noise ratio after ICG signal filtering due to various artifact cancelers (INDBS)

Artifact type	Sample no.	LMS	eN^2^ LMS	SR eN^2^LMS	SE eN^2^LMS	SS eN^2^LMS	N^3^LMS	SR N^3^LMS	SE N^3^LMS	SS N^3^LMS
BWA	Data 1	4.9815	5.8286	7.1277	5.2723	5.0801	8.3075	8.4956	5.7226	5.2454
	Data 2	4.9783	5.8251	7.1626	5.2583	5.1438	8.3059	8.5461	5.6331	5.3726
	Data 3	4.9876	5.8359	7.1035	5.2348	4.9496	8.3081	8.4711	5.5724	5.3241
	Data 4	4.9993	5.8307	7.1964	5.2934	5.1364	8.3317	8.5839	5.6587	5.4356
	Data 5	4.9782	5.8289	7.1732	5.1804	5.1423	8.3184	8.5595	5.7144	5.3976
	Average	4.1884	5.8298	7.1531	5.2480	5.0904	8.3143	8.5312	5.5502	5.3551
EMA	Data 1	4.3813	3.9164	6.8491	3.0812	3.0628	7.4958	7.5570	4.8956	4.7859
	Data 2	4.3905	3.9213	6.9333	3.1025	3.0994	7.4984	7.5823	4.8425	4.6926
	Data 3	4.3802	3.9172	6.7805	3.1583	3.1133	7.4884	7.6118	4.8520	4.7085
	Data 4	4.4016	3.2729	6.8429	3.1693	3.1501	7.5071	7.5523	4.9425	4.6538
	Data 5	4.3912	3.9256	7.1351	3.1979	3.1612	7.4979	7.6445	4.8078	4.6705
	Average	4.3889	3.9216	6.9081	3.1418	3.1173	7.5268	7.5908	4.8701	4.7022
IMA	Data 1	4.0359	3.8136	7.5765	6.2971	4.5841	7.1845	8.4466	7.8773	5.2710
	Data 2	4.0256	3.8371	7.5896	6.2309	4.6642	7.1732	8.4371	7.8473	5.1835
	Data 3	3.9916	3.8096	7.5669	6.2985	4.7363	7.1686	8.4461	7.8435	5.1477
	Data 4	3.9912	3.8140	7.5677	6.2338	4.7059	7.1854	8.4461	7.8435	5.1477
	Data 5	3.9939	3.8079	7.5611	6.2145	4.6768	7.1713	8.3606	7.8658	5.2168
	Average	4.0076	3.8164	7.5723	6.2551	4.6633	7.1766	8.4231	7.8578	5.1884

**Table 2:** Comparison of weight coefficient variations due to various artifact cancelers for data 1

Artifact type	Algorithm	W1	W2	W3	W4	W5	W6	W7	W8	W9	W10
BWA	LMS	0.0559	0.0625	0.0692	0.0759	0.0828	0.0896	0.0965	0.1033	0.1102	0.1170
	e*N* ^*2*^LMS	0.0792	0.0804	0.0817	0.0830	0.0843	0.0857	0.0870	0.0884	0.0898	0.0912
	SRe*N* ^*2*^LMS	0.0435	0.0467	0.0505	0.0560	0.0618	0.0703	0.0805	0.0918	0.1026	0.1177
	SEe*N* ^*2*^LMS	0.0810	0.0835	0.0860	0.0885	0.0911	0.0936	0.0962	0.0988	0.1013	0.1039
	SSe*N* ^*2*^LMS	0.0636	0.0670	0.0705	0.0742	0.0780	0.0820	0.0861	0.0904	0.0949	0.0995
	*N* ^*3*^LMS	0.0606	0.0623	0.0641	0.0660	0.0679	0.0699	0.0719	0.0740	0.0761	0.0783
	SR*N* ^*3*^LMS	0.0604	0.0633	0.0664	0.0699	0.0734	0.0780	0.0829	0.0882	0.0929	0.0987
	SE*N* ^*3*^LMS	0.0788	0.0805	0.0821	0.0838	0.0855	0.0872	0.0890	0.0907	0.0924	0.0941
	SS*N* ^*3*^LMS	0.0694	0.0717	0.0742	0.0767	0.0793	0.0820	0.0848	0.0876	0.0906	0.0937
EMA	LMS	0.1516	0.1338	0.1218	0.1162	0.1162	0.1222	0.1335	0.1497	0.1704	0.1953
	e*N* ^*2*^LMS	0.1040	0.1007	0.0984	0.0971	0.0967	0.0973	0.0987	0.1010	0.1040	0.1079
	SRe*N* ^*2*^LMS	0.2284	0.2284	0.2284	0.2284	0.2284	0.2284	0.2284	0.2284	0.2247	0.4283
	SEe*N* ^*2*^LMS	0.1364	0.1307	0.1251	0.1296	0.1342	0.1390	0.1439	0.1490	0.1543	0.1596
	SSe*N* ^*2*^LMS	0.1166	0.1127	0.1090	0.1134	0.1179	0.1225	0.1273	0.1321	0.1371	0.1422
	*N* ^*3*^LMS	0.1052	0.0979	0.0939	0.0931	0.0950	0.0996	0.1067	0.1163	0.1283	0.1429
	SR*N* ^*3*^LMS	0.1013	0.0877	0.0797	0.0713	0.0705	0.0689	0.0945	0.1087	0.1112	0.1276
	SE*N* ^*3*^LMS	0.0646	0.0501	0.0387	0.0501	0.0672	0.0886	0.1167	0.1540	0.2034	0.2689
	SS*N* ^*3*^LMS	0.1447	0.1304	0.1175	0.1323	0.0953	0.1072	0.1206	0.1357	0.1527	0.1717
IMA	LMS	0.1459	0.1498	0.1538	0.1580	0.1622	0.1665	0.1708	0.1752	0.1795	0.1839
	e*N* ^*2*^LMS	0.0979	0.0989	0.0999	0.1010	0.1021	0.1033	0.1044	0.1056	0.1068	0.1080
	SRe*N* ^*2*^LMS	0.0956	0.0964	0.0978	0.0987	0.1009	0.1014	0.1023	0.1035	0.1041	0.1064
	SEe*N* ^*2*^LMS	0.0530	0.0543	0.0556	0.0570	0.0584	0.0598	0.0613	0.0629	0.0644	0.0660
	SSe*N* ^*2*^LMS	0.0474	0.0540	0.0612	0.0693	0.0781	0.0838	0.0985	0.1101	0.1229	0.1368
	*N* ^*3*^LMS	0.1034	0.1054	0.1074	0.1095	0.1116	0.1138	0.1160	0.1183	0.1205	0.1229
	SR*N* ^*3*^LMS	0.0381	0.0451	0.0553	0.0674	0.0847	0.1081	0.1374	0.1779	0.2322	0.3072
	SE*N* ^*3*^LMS	0.0087	0.0090	0.0094	0.0098	0.0102	0.0106	0.0110	0.0114	0.0118	0.0121
	SS*N* ^*3*^LMS	0.1347	0.4287	0.2743	0.3513	0.4125	0.3187	0.8231	0.5349	0.1250	0.4781

## Discussion

### Baseline-wander artifact (BWA) removal using the DWT adaptive artifact canceller

**Fig. 5:**
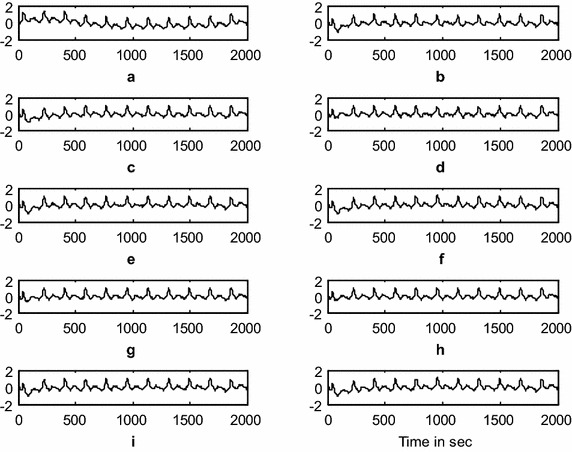
Results of filtering BWA using various AACs (**a**). ICG signal with BWA (**b**). ICG filtering with LMS-updated AAC (**c**). ICG filtering with exponential non-negative LMS (*eN* ^*2*^ *LMS)*-updated AAC (**d**). ICG filtering with SR*eN* ^*2*^ *LMS*-updated AAC (**e**). ICG filtering with SE*eN* ^*2*^ *LMS*-updated AAC (**f**). ICG filtering with SS*eN* ^*2*^ *LMS*-updated AAC (**g**). ICG filtering with normalized non-negative LMS (*N* ^*3*^LMS)-updated AAC (**h**). ICG filtering with SR*N* ^*3*^LMS-updated AAC (**i**). ICG filtering with SE*N* ^*3*^LMS-updated AAC, and (**j**). ICG filtering with SS*N* ^*3*^LMS-updated AAC. (The *x-axis* is the number of samples and the *y-axis* is the signal amplitude in mV)

**Fig. 6:**
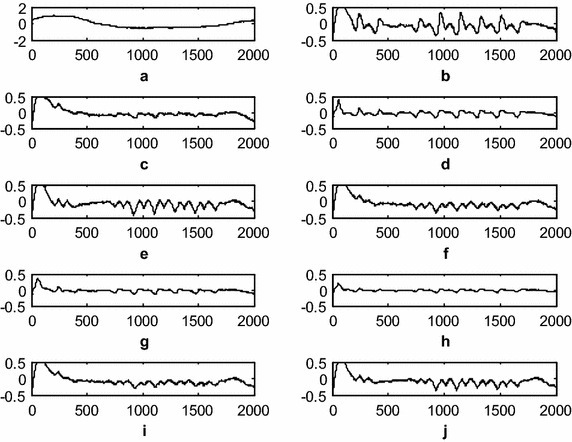
Comparison of the residual noise after BWA filtering (**a**). Original artifact component (**b**). Residual noise after LMS-updated AAC (**c**). Residual noise after exponential non-negative LMS (e*N* ^*2*^LMS)-updated AAC (**d**). Residual noise after SRe*N* ^*2*^LMS-updated AAC (**e**). Residual noise after SEe*N* ^*2*^LMS-updated AAC (**f**). Residual noise after SSe*N* ^*2*^LMS-updated AAC (**g**). Residual noise after normalized non-negative LMS (*N* ^*3*^LMS)-updated AAC (**h**). Residual noise after SR*N* ^*3*^LMS-updated AAC (**i**). Residual noise after SE*N* ^*3*^LMS-updated AAC, and (**j**). Residual noise after SS*N* ^*3*^LMS-updated AAC. (The *x-axis* is the number of samples and the *y-axis* is the residual noise amplitude in mV)

**Fig. 7:**
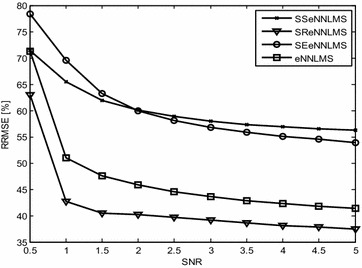
Comparison of the RRMSE of exponential non-negative LMS and its signed versions

**Fig. 8:**
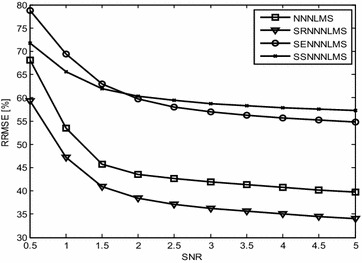
Comparison of the RRMSE of normalized non-negative LMS and its signed versions

This experiment demonstrates the baseline wander artifact cancelation from the ICG signal. The raw ICG signal is input to the DWT-based AAC, as shown in Fig. 1. Using decomposition, DWT constructs a reference signal. This signal is effectively used as a reference signal to the AAC, as shown in the block diagram as *n*_2_. Using feedback from the output *z*(*n*), the algorithm trains *n*_2_ to closely correlate with the artifact component *n*_*1*_ in the input ICG signal *x*(*n*). The SNR and the relative root mean square error (RRMSE) were used to measure the performance of the DWT-based AAC for BWA cancelation. The filtering results of the BWA cancelation are shown in Fig. 5. The results shown in Fig. 5d, g, h are clearer than the results from the other AACs. Figure 6 shows the residual error component after filtering with the various algorithms. The data in Fig. 6h show that the residual error in the case of *SRN*^*3*^*LMS* is less than that of the other algorithms. This is also supported by the SNR table, i.e., from among all of the algorithms, *SRN*^*3*^*LMS* achieves the highest SNR, 8.4956 dBs. The data in Table 2 also show that all of the non-negative algorithms have non-negative weight coefficients when filtering various artifacts. Figures 7 and 8 illustrate the RRMSE (in %) calculated during BWA cancelation. The data in Figs. 7 and 8 show that *eN*^*2*^*LMS*, *N*^*3*^*LMS* and their sign regressor versions perform better the other versions. These algorithms achieve the minimum residual error from among all of the tested algorithms. Finally, from all of the performance measures, *SRN*^*3*^*LMS* is better than the other algorithms with respect to SNR, RRMSE, convergence and computational complexity.

### Electro-muscle artifact (EMA) removal using the DWT adaptive artifact canceller

**Fig. 9:**
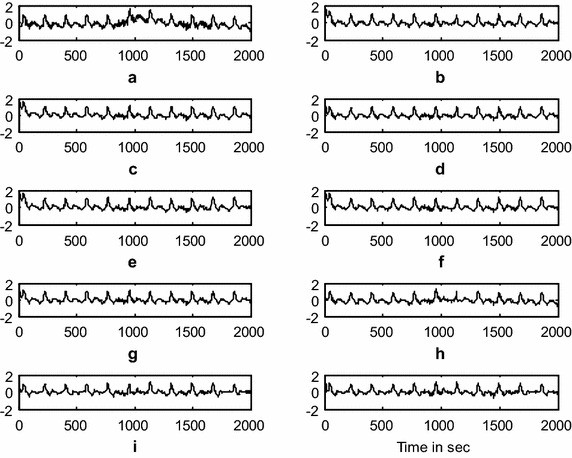
Filtering results of the EMA using various AACs (**a**). Raw ICG with EMA (**b**). ICG filtering with LMS-updated AAC (**c**). ICG filtering with exponential non-negative LMS (e*N* ^*2*^LMS)-updated AAC (**d**). ICG filtering with SRe*N* ^*2*^LMS-updated AAC (**e**). ICG filtering with SEe*N* ^*2*^LMS-updated AAC (**f**). ICG filtering with SSe*N* ^*2*^LMS-updated AAC (**g**). ICG filtering with normalized non-negative LMS (*N* ^*3*^LMS)-updated AAC (**h**). ICG filtering with SR*N* ^*3*^LMS-updated AAC (**i**). ICG filtering with SE*N* ^*3*^LMS-updated AAC, and (**j**). ICG filtering with SS*N* ^*3*^LMS-updated AAC. (The *x-axis* is the number of samples and the *y-axis* is the signal amplitude in mV)

**Fig. 10:**
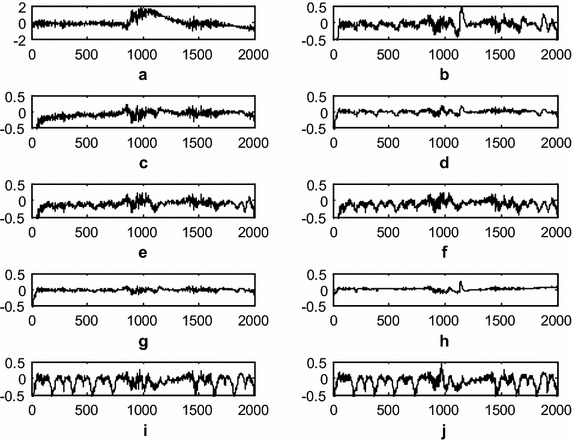
Comparison of the residual noise after EMA filtering (**a**). Original artifact component (**b**). Residual noise after LMS-updated AAC (**c**). Residual noise after exponential non-negative LMS (e*N* ^*2*^LMS)-updated AAC (**d**). Residual noise after SRe*N* ^*2*^LMS-updated AAC (**e**). Residual noise after SEe*N* ^*2*^LMS-updated AAC (**f**). Residual noise after SSe*N* ^*2*^LMS-updated AAC (**g**). Residual noise after normalized non-negative LMS (*N* ^*3*^LMS)-updated AAC (**h**). Residual noise after SR*N* ^*3*^LMS-updated AAC (**i**). Residual noise after SE*N* ^*3*^LMS-updated AAC, and (**j**). Residual noise after SS*N* ^*3*^LMS-updated AAC. (The *x-axis* is the number of samples and the *y-axis* is the residual noise amplitude in mV)

This experiment demonstrates electro-muscle artifact cancelation from an ICG signal. The raw ICG signal is input to the DWT-based AAC, as shown in Fig. 1. The DWT constructs a reference signal using decomposition. This signal is effectively used as a reference signal to the AAC, as shown in the block diagram as *n*_2_. Using feedback from the output *z*(*n*), the algorithm trains *n*_*2*_ to closely correlate it with the artifact component *n*_*1*_ in the ICG input signal *x*(*n*). The SNR is used to measure the performance of the DWT-based AAC for EMA cancelation. The filtering results of the EMA cancelation are shown in Fig. 9. The results shown in Fig. 9d, g, h are clearer than the results from the other AACs. Figure 10 shows the residual error component after filtering with various algorithms. The data in Fig. 10h show that the residual error in the case of *SR N*^*3*^*LMS* is less than that for the other algorithms. This conclusion is supported by the SNR table; among all of the algorithms *SR N*^*3*^*LMS* achieves the highest SNR of 7.5570 dBs. The data in Table 2 also show that all of the non-negative algorithms use non-negative weight coefficients when filtering various artifacts. Finally, to summarize the performance measures, is the results show that *SR N*^*3*^*LMS* is better than the other algorithms with respect to SNR, RRMSE, convergence and computational complexity.

### Impedance mismatch artifact (IMA) removal using the DWT adaptive artifact canceller

**Fig. 11:**
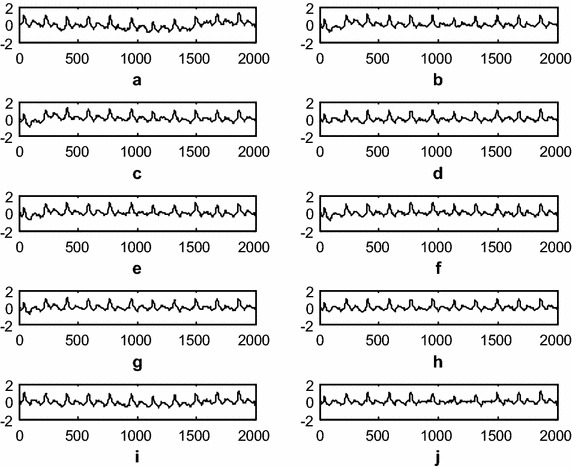
Filtering results for IMA from the various AACs (**a**). ICG signal with IMA (**b**). ICG filtering with LMS-updated AAC, (**c**). ICG filtering with exponential non-negative LMS (e*N* ^*2*^LMS)-updated AAC (**d**). ICG filtering with SRe*N* ^*2*^LMS-updated AAC (**e**). ICG filtering with SEe*N* ^*2*^LMS-updated AAC (**f**). ICG filtering with SSe*N* ^*2*^LMS-updated AAC (**g**). ICG filtering with normalized non-negative LMS (*N* ^*3*^LMS)-updated AAC (**h**). ICG filtering with SR*N* ^*3*^LMS-updated AAC (**i**). ICG filtering with SE*N* ^*3*^LMS-updated AAC, and (**j**). ICG filtering with SS*N* ^*3*^LMS-updated AAC. (The *x-axis* is the number of samples and the *y-axis* is the signal amplitude in mV)

**Fig. 12:**
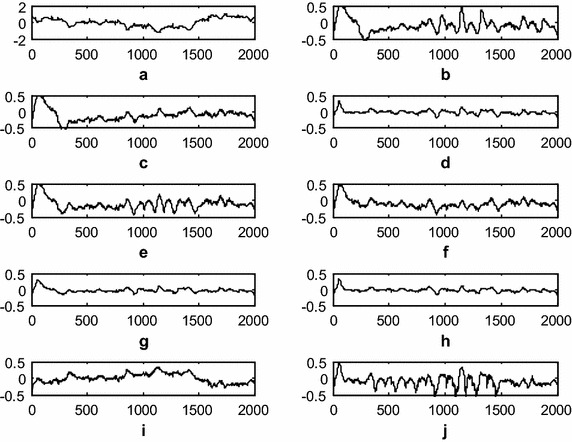
Comparison of the residual noise after IMA filtering (**a**). Original artifact component (**b**). Residual noise after LMS-updated AAC (**c**). Residual noise after exponential non-negative LMS (e*N* ^*2*^LMS)-updated AAC (**d**). Residual noise after SRe*N* ^*2*^LMS-updated AAC (**e**). Residual noise after SEe*N* ^*2*^LMS-updated AAC (**f**). Residual noise after SSe*N* ^*2*^LMS-updated AAC (**g**). Residual noise after normalized non-negative LMS (*N* ^*3*^LMS)-updated AAC (**h**). Residual noise after SR*N* ^*3*^LMS-updated AAC (**i**). Residual noise after SE*N* ^*3*^LMS-updated AAC, and (**j**). Residual noise after SS*N* ^*3*^LMS-updated AAC. (The *x-axis* is the number of samples and the *y-axis* is the residual noise amplitude in mV)

This experiment demonstrates impedance mismatch artifact cancelation from the ICG signal. The raw ICG signal is input to the DWT-based AAC, as shown in Fig. 1. The DWT constructs a reference signal using decomposition. This signal is effectively used as a reference signal for the AAC, as shown in the block diagram as *n*_2_. Using feedback from the output *z*(*n*), the algorithm trains *n*_2_ to closely correlate with the artifact component *n*_*1*_ in the input ICG signal *x*(*n*). The SNR is used to measure the performance of the DWT-based AAC for EMA cancelation. The filtering results of the EMA cancelation are shown in Fig. 11. The results shown in Fig. 11d, g, h are clearer than the results for the other AACs. Figure 12 shows the residual error component after filtering with the various algorithms. The data in Fig. 12h show that the residual error in the case of *SR N*^*3*^LMSis less than that of the other algorithms. This also is supported by the SNR table, i.e., among all of the algorithms, *SR N*^*3*^LMS achieves the highest SNR at 8.4466 dBs. The data in Table 2 also show that all of the non-negative algorithms have non-negative weight coefficients when filtering the various artifacts. Finally, all of the performance measures indicate that *SRN*^*3*^LMSis better than the other algorithms with respect to SNR, RRMSE, convergence and computational complexity.

## Conclusion

This paper presents a new technique for enhancing ICG signals for telecardiology applications. The primary feature of the proposed adaptive artifact canceller is that it does not require a reference signal. The proposed model itself constructs a reference signal using DWT decomposition. This constructed signal is used to train the filter weight coefficients for the noise cancelation process. To avoid computational divergence caused by negative weights during abnormal heart conditions, we use a non-negative LMS algorithm and its variants. Based on this constraint, we developed various non-negative algorithms, that is, *eN*^*2*^*LMS*, *SR eN*^*2*^*LMS*, *SE eN*^*2*^*LMS*, *SS eN*^*2*^*LMS*, *N*^*3*^*LMS*, *SRN*^*3*^*LMS*, *SEN*^*3*^*LMS* and *SS N*^*3*^*LMS*. Among these algorithms, the sign regressor-based algorithms require fewer multiplications and achieve better convergence because of an additional normalization factor used in the signum function. Additionally, the convergence characteristics of the sign regressor version are slightly inferior to its unsigned version. Therefore, with respect to the SNR shown in Table 1, the weight coefficients shown in Table 2, the filtering results, the residual error, the RRMSE curves and the computational complexity, the results show that the *SRN*^*3*^*LMS*-based AAC performs better than the other algorithms. Hence, this DWT-based AAC is well-suited for noise cancelation in remote health care monitoring systems.
