# Erratum to: Efficient reference-free adaptive artifact cancellers for impedance cardiography based remote health care monitoring systems

**DOI:** 10.1186/s40064-016-2738-8

**Published:** 2016-07-14

**Authors:** Madhavi Mallam, K. Chandra Bhushana Rao

**Affiliations:** Department of Electronics and Communication Engineering, Jawaharlal Nehru Technological University, Kakinada, AP 533003 India; Department of Electronics and Communication Engineering, JNTUK, University College of Engineering, Vizianagaram, AP 535002 India

## Erratum to: SpringerPlus (2016) 5:770 DOI 10.1186/s40064-016-2461-5

Upon publication of the original article (Mallam and Bhushana Rao 2016), it was noticed that K. Chandra Bhushana Rao’s name was incorrectly written as ‘K. Chandra Bhutan Rao’. This has now been updated.
